# A Novel Method for Estimating Free Space 3D Point-of-Regard Using Pupillary Reflex and Line-of-Sight Convergence Points

**DOI:** 10.3390/s18072292

**Published:** 2018-07-15

**Authors:** Zijing Wan, Xiangjun Wang, Kai Zhou, Xiaoyun Chen, Xiaoqing Wang

**Affiliations:** 1 State Key Laboratory of Precision Measurement Technology and Instruments, Tianjin University, No. 92 Weijin Road, Nankai District, Tianjin 300072, China; zjwan@tju.edu.cn (Z.W.); zhoukrwx@163.com (K.Z.); 2 MOEMS Education Ministry Key Laboratory, Tianjin University, No. 92 Weijin Road, Nankai District, Tianjin 300072, China; cxy941107@tju.edu.cn (X.C.); tjuwxq@tju.edu.cn (X.W.)

**Keywords:** eye tracking, 3D Point-of-Regard, pupillary reflex, head-mounted device

## Abstract

In this paper, a novel 3D gaze estimation method for a wearable gaze tracking device is proposed. This method is based on the pupillary accommodation reflex of human vision. Firstly, a 3D gaze measurement model is built. By uniting the line-of-sight convergence point and the size of the pupil, this model can be used to measure the 3D Point-of-Regard in free space. Secondly, a gaze tracking device is described. By using four cameras and semi-transparent mirrors, the gaze tracking device can accurately extract the spatial coordinates of the pupil and eye corner of the human eye from images. Thirdly, a simple calibration process of the measuring system is proposed. This method can be sketched as follows: (1) each eye is imaged by a pair of binocular stereo cameras, and the setting of semi-transparent mirrors can support a better field of view; (2) the spatial coordinates of the pupil center and the inner corner of the eye in the images of the stereo cameras are extracted, and the pupil size is calculated with the features of the gaze estimation method; (3) the pupil size and the line-of-sight convergence point when watching the calibration target at different distances are computed, and the parameters of the gaze estimation model are determined. Fourthly, an algorithm for searching the line-of-sight convergence point is proposed, and the 3D Point-of-Regard is estimated by using the obtained line-of-sight measurement model. Three groups of experiments were conducted to prove the effectiveness of the proposed method. This approach enables people to obtain the spatial coordinates of the Point-of-Regard in free space, which has great potential in the application of wearable devices.

## 1. Introduction

Human vision is the most important sense of human beings. Our eye movement also contains a great deal of visual attention and emotional information. Nowadays, many eye tracking methods have been developed to analyze the behavior of human eyes. These methods enable computers to understand human visual attention. Based on the gaze information, the human-computer interaction function can then be realized.

Existing state-of-the-art methods can be effective in a specific application environment, but it is still a relatively difficult problem to extract the depth perception of the observer from human eye movement information. In other words, it is still a challenge to estimate the three-dimensional coordinates of human’s gaze points in free space by using only eye features. Therefore, a novel method for estimating free space 3D Point-of-Regard (PoR) is proposed in this paper by using the spatial positions of the eye features (i.e., inner eye corner point and pupil edges). Firstly, a simplified line-of-sight measurement geometry model combining the properties of the pupillary accommodation reflex and the convergence of the line-of-sight into one point is proposed. Secondly, a specific calibration process is presented to calibrate line-of-sight convergence points. The PoR estimation method described in this paper can realize the measurement of the spatial coordinates of binocular gaze point, effectively calculate the spatial coordinates of the PoR and reflect the depth perception of human vision. In addition, experiments are carried out to validate the effectiveness and accuracy of the proposed method.

Before introducing our method, two basic properties of the human eye are given. Pupillary reflex is the effect of enlarging or reducing the pupil after receiving external stimulus. In eye movement research, pupillary light reflex and pupillary accommodation reflex are two common phenomena. The significance of pupillary accommodation reflex in human vision is to make the human eye clear images of objects with different distances. When a person looks at an object in a closer distance, it will reflectively make the pupil smaller. Pupillary reduction can decrease spherical aberration, simultaneously decrease the view range of the cornea, and also decrease the astigmatism caused by irregular curvature of the cornea. Moreover, the human eye vision system has strong adjusting ability, and can present inverted real images on the retina when observing objects with different distances, so that light rays entering the eye converge into a single point in the eyeball. In this paper, we call the spatial location of the converging point of light entering the pupil the line-of-sight convergence point.

The pupillary reflex of human vision is stable when human eyes are looking at non-specific objects for a not too long period of time, and it is not affected by other factors except environmental illumination transformation. Therefore, the proposed method uses this characteristic to correlate the pupil size with the distance of the observed object. The head-mounted gaze tracking device can be easily installed in free space, and the pose of human body or head is usually obtained by Simultaneous Localization and Mapping (SLAM) technology or inertial sensors, so this paper focuses on the estimation of the line-of-sight and the 3D PoR.

In this paper, we estimate the line-of-sight direction of both eyes based on the spatial positions of the eye features (i.e., inner eye corner point and pupil edges) calculated from the images captured by the stereo cameras. A line-of-sight measurement geometry model combining the properties of the pupillary accommodation reflex and the convergence of the line-of-sight into one point is proposed. This model is a simplified geometry-model, and this paper also proposes a specific calibration process for calibrating line-of-sight convergence points. The contributions of this paper are:

1. A novel stereo-camera based gaze tracking system is proposed to accurately track a person’s eyes. This gaze tracking system is equipped with a pair of novel light source and semi-transparent mirrors. This specific device aims to capture clearer human eye images without affecting the natural observation of the human eye. This paper also describes the key parameters of the stereo cameras and the stereo vision model of these cameras. The form of this gaze tracking system has the potential to be transformed into a head-mounted gaze tracking device.
2. This paper demonstrates a simplified geometry-model. The key feature of this model is that the light entering the eye will converge in the eye at a line of sight convergence point. In this model, the pupil edge and the inner eye corner are the utilized features. The spatial coordinates of these eye features can be solved by using stereo cameras, thus we can directly use the spatial coordinates of these features to determine the spatial coordinates of the line-of-sight convergence point. This model matches with a relatively simple calibration process, and it achieves the calculation of the spatial intersection point of the binocular line-of-sight.
3. This paper also proposes a calibration process. A specific calibration target is placed in several different positions and a person is allowed to watch the four calibration points on the target. These calibration data are then processed by a search approach to obtain the line-of-sight convergence point, and the relationship between the pupil size and the line-of-sight convergence point position is solved. This calibration process can become a general framework of the system calibration method for the free-space gaze tracking system.

## 2. Related Works

The gaze estimation method based on visible light camera is the main content of current research on gaze estimation technology. Earlier research looked forward to the development prospect of gaze tracking technology in the human-computer interaction and medical health [1]. Moreover, the technical framework proposed by the forward-looking research in [2] is already close to the current research. However, with the improvement of imaging quality of image sensors and more powerful micro-computers, the technical maturity of recent commercial eye tracking technologies can reach a very high level. Early application studies laid a foundation for the current hot topics such as intelligent driving, and the contributions combining eye tracking technology have been successfully applied to fields such as driver’s fatigue detection and disability assistance [3,4]. Ji, Hansen, et al. have carried out in-depth research work on the gaze estimation [5,6] and have concluded two main types: methods based on geometry and appearance.

Geometry-based methods rely on the detection of local features and require one or more models to map local features to line-of-sight parameters. Almost all methods require a calibration process to establish mapping and require high-resolution images. The pupil-cornea reflection method, which relies on near-infrared illumination and a camera, can be applied to both a remote camera and head-mounted types of devices to estimate the line-of-sight direction and the Point-of-Regard from the pupil center and cornea reflection.

In order to obtain higher 3D gaze estimating accuracy and enable the system to be applied in an environment where the head moves freely, the deployment of multiple light sources and multiple cameras is an easy solution to consider. A few research also used multiple types of cameras and proposes concerned calibration methods, and the error of line-of-sight estimation was reduced to within one degree [7,8]. The application of multiple cameras and light sources in the system can improve the overall method’s environmental adaptability and line-of-sight estimation accuracy. One of the research focus of these work was still to deal with spot noise and eyelid occlusion, such as the work presented by [9]. Another research focus was on how to calibrate a specific system more simply [10,11,12,13], and these listed methods can make the gaze tracking system use a simple calibration process, little personal calibration, and the ability to work steadily. Besides, the research on the calibration process of a gaze tracking system has always been the focus of this research.

The appearance-based method establishes a direct mapping from the eye or head image to gaze estimating parameters. The advantage of this kind of methods is that they do not need to consider the detection or tracking of local facial features, and these methods have potential to support the gaze estimation in low-resolution images. Earlier approaches have begun to use machine learning to train gaze estimation models or to reduce errors [14,15]. In recent years, the learning-based methods for appearance-based gaze estimation have received a lot of interest, Random Forest (RF), k-Nearest Neighbors (kNN), Support Vector Regression (SVR), etc. Experimental results showed that these algorithms could be applied to gaze estimation for natural images, and support stability and efficiency in practical applications [16,17]. According to [18], it was appropriate to improve the effectiveness of the learning-based method by using training a dataset which was combined the 3D model of the human eye region, and the gaze direction error was lower than 10 degrees. Although the learning-based methods have strong advantages in scene adaptability, their gaze direction accuracy is still far lower than that of the geometry-based methods.

A clustering approach was proposed to estimate the head movement. And this approach used human-computer interacting trajectories as training data in the local linear embedding of samples prior to clustering [19]. Later on, the authors added sub-pixel alignment and blink detection to the improvements, and they claimed that this kind of method could effectively deal with the situation under slight head movement or static states [20]. George and Routray [21] proposed to use fast convolution and boundary ellipse fitting to quickly locate the iris center, which ensured low-resolution gaze tracking with sufficient accuracy. Additionally, the method described in [22] also extracted pupil and iris features and used the Maximum a Posteriori (MAP) framework to ensure tracking accuracy. This work could also ensure high accuracy in low-resolution images.

Next, we give a brief discussion on the different structures of the gaze tracking system and their respective advantages and disadvantages. The remote camera-based methods usually consider head pose estimation and gaze estimation at the same time, which can be carried out independently, and recent methods can achieve good accuracy. While the head-mounted camera-based method can obtain clear eye images, which needs to rely on extra image sensors or pose measurement tools to estimate the head’s motion. In addition, these approaches can be freely installed. Head-mounted gaze trackers are usually equipped with at least one eye camera to track eye movements [23], and near-infrared light source. The near-infrared light source can not only provide reflection spots as the main feature, but also improve the contrast of eye images. New devices proposed in recent years usually use multiple cameras [24], including at least one scene camera. Near-eye formed gaze tracker also has advantages in capturing clear eye images [25].

The methods using RGB-D cameras are able to effectively reconstruct facial features to achieve gaze estimation [26,27,28]. Currently, consumer-grade RGB-D cameras can reconstruct head posture changes well and ensure the resolution of the eye region images is sufficient at the same time. The method based on a depth camera still has potential, under the condition that the object to be measured is completely unconstrained. However, in order to achieve low cost and easy deployment, the gaze tracking device based on visible light camera is still the most ideal implementation.

Through the investigation of gaze tracking techniques, we find that these existing techniques can extract the direction of line-of-sight very accurately. However, because most of them belong to the construction of a relationship model between the human eye information and the observed object in a fixed position, it is difficult for most of the existing methods to obtain accurate 3D gaze information. Therefore, recent 3D gaze tracking focuses on how to obtain the depth information of the PoR. Takemura et al. [23] combined the observed object image and the pupil vector, estimated the 3D coordinates of the PoR by Delaunay triangle approximation and estimated the pose of the head using the scene camera and attitude sensor. The method attempted to use the corner feature points extracted by the scene camera to match the intersection point of the line-of-sight and the scene and estimated the coordinates of the Point-of-Regard. However, this method relied on the feature points extracted from the images captured by the scene camera.

Li, Zhang and Webb [29] proposed a method that could allow the gaze point to control the movement of a robot arm to a specified position and proposed a method to calculate the spatial line-of-sight intersection point. This method has higher positioning accuracy in a smaller spatial range and does not limit the movement of the head. Fusing pupil features and the multi-camera view geometry is also a method to calculate the coordinate of the Point-of-Regard [30]. The calibration process of this method is simple and effective. Its advantage is that it does not need to assume a specific complex model of the eyes. Ferhat and Vilariño [31] conducted a review of the existing state-of-the-art visible light camera-based gaze tracking approaches. They concluded that focusing on the use of new features might be the focus of work to improve the performance of the gaze tracker. The studies analyzed above all attempts to solve the spatial coordinates of the PoR, but there are still problems such as the need for specific scenes or markers.

## 3. Methods

**Figure 1:**
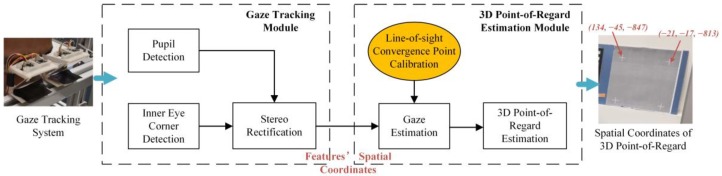
The architecture of the 3D Point-of-Regard estimation method.

In order to achieve the 3D PoR estimation when a user is naturally looking at the object, two major works are included in the paper: (1) a customized gaze estimation system and the approach of the eye features extraction and the spatial localization, this part of work would be demonstrated in Section 2, Section 3.1 and Section 3.2) system calibration process and 3D PoR estimation, this part of work would be demonstrated in Section 3.3. The customized experimental system and the method architecture is demonstrated in Figure 1.

The customized system uses two pairs of cameras to obtain the spatial coordinates of the features of the left and right eyes. The accurate extraction and spatial localization of the features of the eyes can make the center of the pupil and the inner eye corner as the basis for solving the spatial coordinate of 3D PoR.

The line-of-sight estimation uses a simplified geometry-model to solve the parameters of the spatial line where the line-of-sight are located. Besides the basic model of the gaze estimation, we mainly describe a system calibration process in Section 3.3 to obtain the parameters of the line-of-sight geometry-model. The calibration process mainly aims to the search of the line-of-sight convergence point and relate the corresponding pupil size. After obtaining the fitting equation of the line-of-sight convergence point, the 3D PoR estimation result can be obtained by calculating the nearest point of the two spatial lines of the binocular line-of-sight.

### 3.1. Customized Gaze Estimation System

According to previous research results, gaze points can be obtained directly from the eye image. The gaze tracking system based on visible light cameras needs to use cameras and light sources with different structures in order to satisfy different methods. Usually, the head-mounted gaze tracking system needs to meet three conditions in its design:

1. Ensure that users have a large field of view;
2. The cameras avoid or minimize the influence of environmental illumination;
3. Head-mounted devices should be as light as possible.

Therefore, the gaze tracking device proposed in this paper mainly considers these three conditions when designing and proposes a more compact line-of-sight tracking device based on low-cost components.

**Figure 2:**
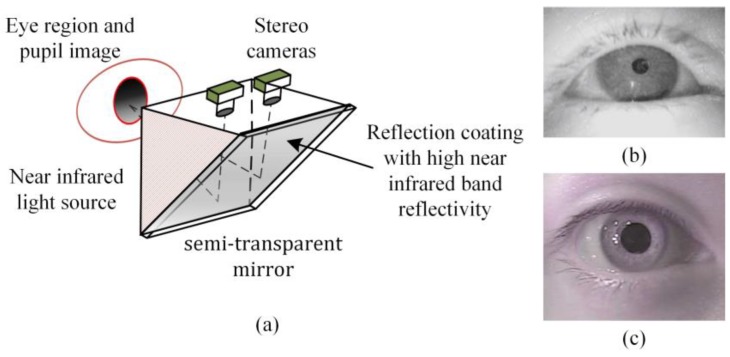
(**a**) The gaze tracker module of the left eye, and the shape and position of the stereo cameras, the light source, and the semi-transparent mirror are drawn in the figure; (**b**) the eye image captured by a Dikablis gaze tracker (Ergoneers GmbH, Manching, Germany); and (**c**) the eye image captured by proposed gaze tracker, the pupil and the eye corners can be identified clearly.

The structure of the gaze estimation system in this paper is based on multiple view geometry. This gaze estimation method in 3D space provides users with a wide field of view. Since the mirror is placed at 45 degrees, the cameras can capture images from the position where is equivalent to the directly front of the eyes. Therefore, the system is more ideal than some common gaze tracking systems (e.g., Tobii Glasses and Dikablis Glasses) in extracting eye features. Since the method of gaze estimation relies on clear pupil images, we also set a near-infrared light source to obtain dark pupil images. The light source is made into a triangular light-emitting panel, and then two pieces of such light-emitting panels are placed between the two cameras. The illustration of the gaze tracker of the left eye is shown in Figure 2. This design enables the light source to evenly illuminate the human eye area, while we reduce the brightness of the light source as far as possible so as to reduce the stimulation of the light source to the eyes.

**Figure 3:**
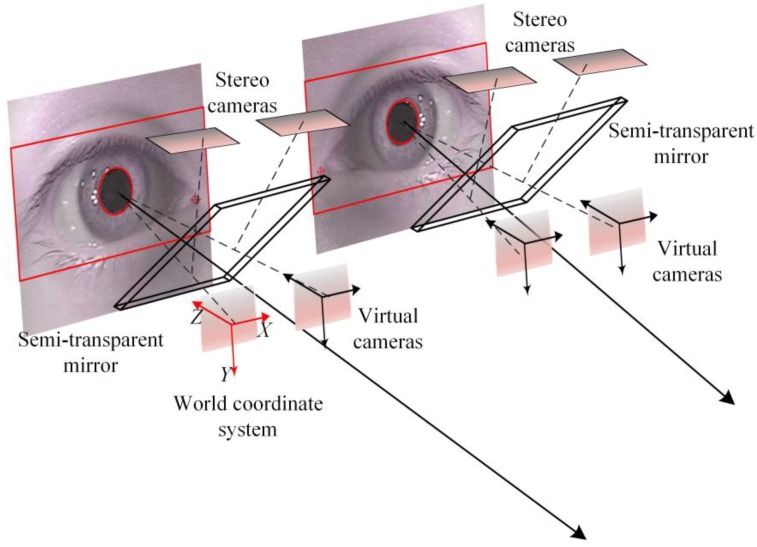
The gaze estimation system with coordinate definition.

The gaze estimation model is formed by four cameras, and these four cameras are divided into two groups; each group of cameras focuses on one of the eyes. We designed a special semi-transparent mirror. Its reflective surface for the eye has high near-infrared band reflectivity, while the visible light band transmittance is higher than 70%. This enhances the illumination effect of the near-infrared light source while reducing the interference of ambient light with the camera imaging. This semi-transparent mirror is installed in front of the eye cameras. This setup can effectively reduce the impact of ambient light noise on the process of gaze estimation. The illustration of the gaze estimation system with coordinate definition is shown in Figure 3.

We fix the relative position of the cameras in the system with the calibration target. After the installation of the gaze estimator, the stereo cameras would be calibrated by a common camera calibration method, but what is special is that the calibration is based on the four cameras’ virtual images. In this paper, we call them virtual cameras. In fact, this does not change the property of the imaging process, but it is important because it determines the definition of the world coordinate system. The world coordinate system coincides with the camera coordinate system of the leftmost camera as shown in Figure 2. The advantage is that it is not necessary to calibrate the relationship between the real camera and the reflector in a difficult way, and the world coordinate system based on the virtual camera can make the obtained data more intuitive.

**Figure 4:**
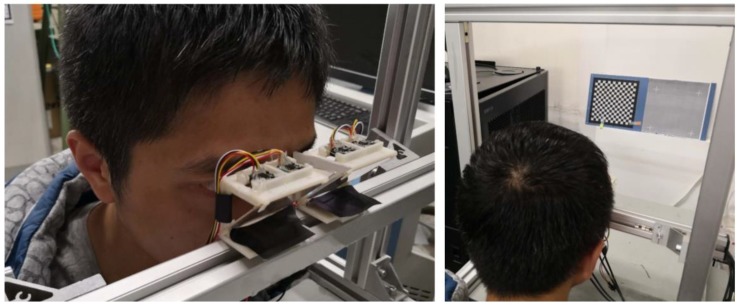
Customized gaze tracking system, with four cameras in 640 × 580 resolution, which can be converted into a head-mounted device.

This device is divided into left and right modules, and the size of a single module is 50 mm × 35 mm × 40 mm, and the distance between the two modules can be adjusted according to the pupil distance of different persons. Cameras are placed vertically, and the baseline of a pair of cameras is 22 mm. The focal length of a single camera is 3.6 mm, and the resolution of single camera is 640 × 580 pixels. The whole gaze tracking system proposed in this paper is shown in Figure 4.

### 3.2. Eye Features for Gaze Estimation

In this paper, we choose the pupil and the inner eye corner to estimate the gaze direction. These two features are stable and obvious in the image. On the other hand, assuming that the center of the pupil can represent the center of the field of view, and there is a line-of-sight convergence point in the eyeball, if we can determine the spatial position of the center of the pupil and the line-of-sight convergence point, we can easily calculate the spatial line-of-sight.

**Figure 5:**
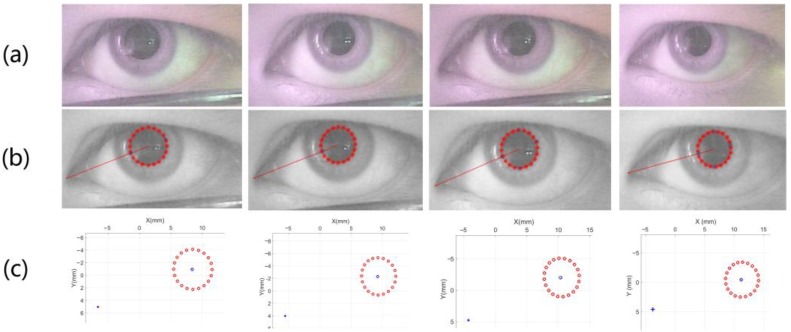
(**a**) represents the change of the pupil size when watching objects at different distances; (**b**) represents the extraction of the pupil and inner eye corner; and (**c**) shows the spatial coordinates of the inner eye corner and pupil edge.

According to [32], under fixed light intensity, the eye pupil changes from large to small with the observed object from far to near. Therefore, this paper assumes that the illumination condition of the scene is stable, which makes the change of pupil size reflect the distance of the object observed by the human eye. According to [33], the depth perception of human eyes can affect the three-dimensional scene vision in the brain. How to establish a method of line-of-sight measurement to obtain the depth perception of eyes is the main work of this paper. This enables the method to adapt to most of the use environments, while the only thing that needs to be limited is the fixed environmental illumination condition. The results of feature extraction and spatial coordinates solving are shown in Figure 5. In applications, it is not possible to observe a luminous object, such as a screen. This is due to the natural contraction of the pupil when the eyes observe luminescent objects, so that the relationship between pupil size and the distance from the observation distance is invalid. However, this method can be applied when the position of the head and screen is fixed.

According to [34], nearly all of the state-of-the-art pupil detection and pupil edge extraction approaches were also based on edge detection and ellipse fitting, because of their efficiency and accuracy. An approach for accurately extracting the pupil edge is proposed in this paper. This method uses the adaptive gray gradient edge extraction to extract the pupil edge, and then uses these points by using the RANSAC-based ellipse fitting to obtain the pupil edge in the image. This method considers how to deal with the problem of bright spot and eyelid occlusion when designing an eye-tracking device. First, a horizontal and vertical projective operation is used to locate the human eye area from the image. Usually, we can obtain clear pupil images, but when eyeball is fast moving or occluded by eyelid, the images are not good enough. At this time, image segmentation using the adaptive threshold segmentation method, such as the Otsu method, is not effective. Therefore, the reason why we analyze the blurred image is that the eye is turning far away from the light source, or when the current image frames are taken, the eyeball is moving rapidly.

For an image I(x,y), the size is m×n, we firstly calculate the image gradient amplitude map, $G(x,y)=\sqrt{{\left(\frac{\partial F}{\partial x}\right)}^{2}+{\left(\frac{\partial F}{\partial y}\right)}^{2}}$. Then, the gradient amplitude near the center and the four corners of the image is set to 0, and the gradient amplitude map is divided into 3 × 3 blocks. Then the gradient value which is above the average of the gradient values in each block is extracted. After completing the steps above, traversing points with a gradient value greater than 0, and the gradient value near the bright spot is first set to be 0, obtaining *G(x*, *y).* The resulting map *GG(x*, *y)* is calculated according to the function:

(1) $$\begin{array}{l} g(x,y)\in G(x,y),x=1,2,\cdots ,m,y=1,2,\cdots ,n\\ GG(x,y)=\left\{ \begin{array}{l} 1,\sum_{p=x-1}^{x+1}\sum_{q=y-1}^{y+1}g(p,q)>0.6\times \frac{\sum_{x=1}^{m}\sum_{y=1}^{n}\left(\sum_{t=x-1}^{x+1}\sum_{s=y-1}^{y+1}g(t,s)\right)}{6\times m\times n}\\ 0 \end{array}\right. \end{array}$$

After obtaining the pupil edge point map *GG(x*, *y)*, we use two-step ellipse fitting to obtain the pupil ellipse. In this process, we firstly use RANSAC-based ellipse fitting, and then compare the distances between fitting points and the nearest points on the fitting ellipse. The fitting parameter equation is:

(2) $$\left(\begin{array}{l} x\\ y \end{array}\right)=\left(\begin{array}{c} X_{0}\\ Y_{0} \end{array}\right)+\left(\begin{array}{cc} \cos\alpha & -\sin\alpha \\ \sin\alpha & \cos\alpha \end{array}\right)\left(\begin{array}{c} A\cos\theta \\ B\sin\theta \end{array}\right)$$

Among them, *(x*, *y)* is the image coordinate of the fitting points, *(X*_0_, *Y*_0_*)* is the image coordinate of the center of the ellipse. The *α* is the rotation angle of the major axis relative to the transverse axis of the image, *θ* is the rotation angle of the fitting point on the ellipse, and *A* and *B* are the major axis and the minor axis of the ellipse, respectively.

**Figure 6:**
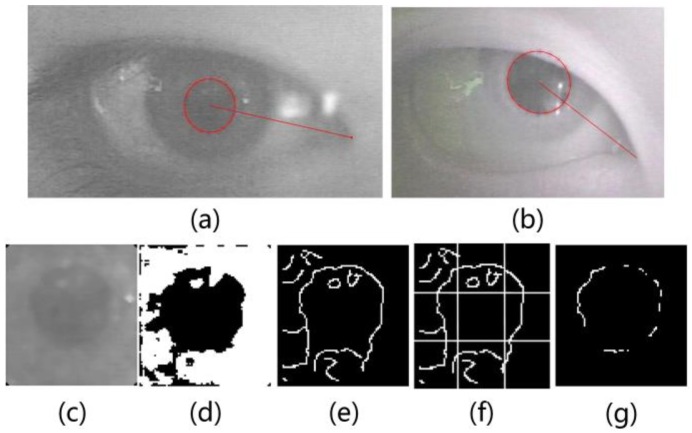
(**a**) represents the extraction of pupil when image with low contrast; (**b**) represents the extraction of the pupil when the pupil is occluded; (**c**) represents the pupil area; (**d**) represents the result of the Otsu method; (**e**,**f**) represent the preliminary result of pupil edge detection; and (**g**) represents the detection result of the pupil edge.

The point is set to 0 when the distance between the fitting point and the closest point on the ellipse is greater than the 1/8 of the length of major axis. After steps above, the final pupil ellipse is obtained by the least square method-based ellipse fitting, and the fitting parameter equation of this step is the same as Equation (2). As shown in Figure 6, the method can obtain an accurate pupil edge.

**Figure 7:**
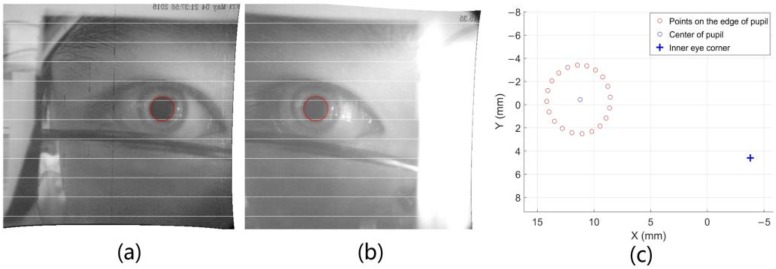
(**a**,**b**) represent the images of binocular cameras after epipolar rectification; and (**c**) represents the detected pupil and inner eye corner.

The spatial position and size of the pupil are matched by the epipolar constraint, and the obtained matching points are used to obtain the spatial circle of the pupil by using the least square fitting 3D circle. The left and right images after epipolar rectification are shown in Figure 7a,b and the solving result of the spatial coordinates of the pupil edge is shown in Figure 7c.

In this paper, we adopt an inner eye corner extraction method based on multi-scale Harris corner detection [35]. As mentioned above, the area of the eye is obtained by a horizontal and vertical projective operation, and then the inner eye corner region can be easily located by threshold segmentation. The multi-scale Harris corner detection and the weight distribution of different scales are used to find the exact inner eye corner point. After obtaining the position of the inner eye corner of each image, the spatial coordinates of the corner points of the left and right eyes can be solved by stereo matching.

**Figure 8:**
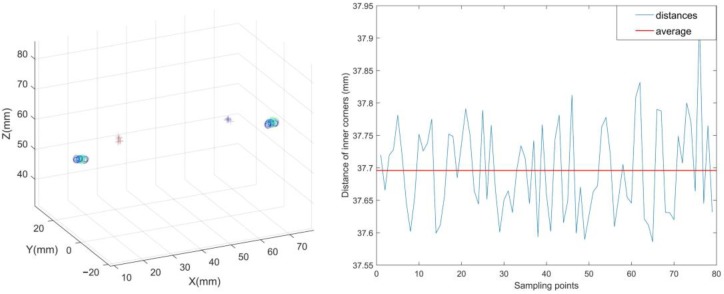
The (**left**) graph represents the extraction result of inner eye corner and centers of eyes in 80 replications. The (**right**) graph shows the distances from the left to right eye corners in the experiment.

In a random experiment, we collected 80 groups of images to test the stability of the inner eye corner feature. The distance between the left and right inner corners is the benchmark and length changes of these distances are compared. The experimental result is shown in Figure 8, and the standard deviation is less than 0.10 mm. Through this experiment, we can obtain the stable inner eye corners by our method, and it can be proved that the inner eye corner is available as the reference feature for the estimation of the Point-of-Regard.

### 3.3. Estimation of the 3D Point-of-Regard and the System Calibration

The location of the line-of-sight convergence point is determined by the location of the pupil center and the shape of the calibration object. This section proposes a 3D Point-of-Regard estimation model and the corresponding calibration process. Using a calibration target with free placement, the spatial coordinates of the line-of-sight convergence points can be estimated.

According to Section 3.2, the pupil size and the center of the pupil have been calculated by stereo rectification. According to the 3D Point-of-Regard estimation model, we present a calibration process to find a pair of line-of-sight convergence points of binocular eyes, and the spatial coordinates of the eye features extracted during calibrating are the key features when searching the line-of-sight convergence points. The calibration process is performed by the person who would observe a specific target at different distances several times. After the calibration process, we also need to integrate the spatial coordinates of the line-of-sight convergence points to the same coordinate system. In this paper, a spatial coordinate alignment approach based on the inner eye corner vector is proposed. Finally, a polynomial fitting equation is used to determine the relation equation between the line-of-sight convergence points and the pupil size.

**Figure 9:**
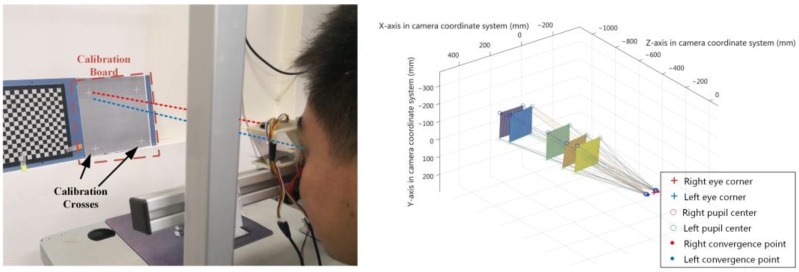
The spatial coordinates of the Point-of-Regard in five calibration steps. The (**left**) graph shows a real picture when using the gaze tracking system. The (**right**) graph shows the system calibration result.

In Section 3.3.1, we will describe the entire gaze estimation model in detail and analyze the search method when calibrating the line-of-sight convergence point. In Section 3.3.2, the spatial coordinate alignment approach based on the inner eye corner vector is demonstrated and also the coordinate transformation process. The result of one calibration process is shown in Figure 9.

#### 3.3.1. Calibration Process

The design of the calibration board is given at the beginning. We customize a gray colored calibration board. There are four white crosses in the four corners, and the distances between each two crosses are 150 mm. The person has to look at the four crosses of the calibration board. In the whole calibration process, this calibration board moves five times, so the camera system should capture the eye images 20 times. The calibration board should be placed in any five different distances ranging from 40 cm to 120 cm, and the calibration board needs to be basically straightened to the gaze tracking system. The experiment needs to keep the relative position of the head and the system fixed, and the four crosses of the calibration board need to fully attract the visual attention of the user.

**Figure 10:**
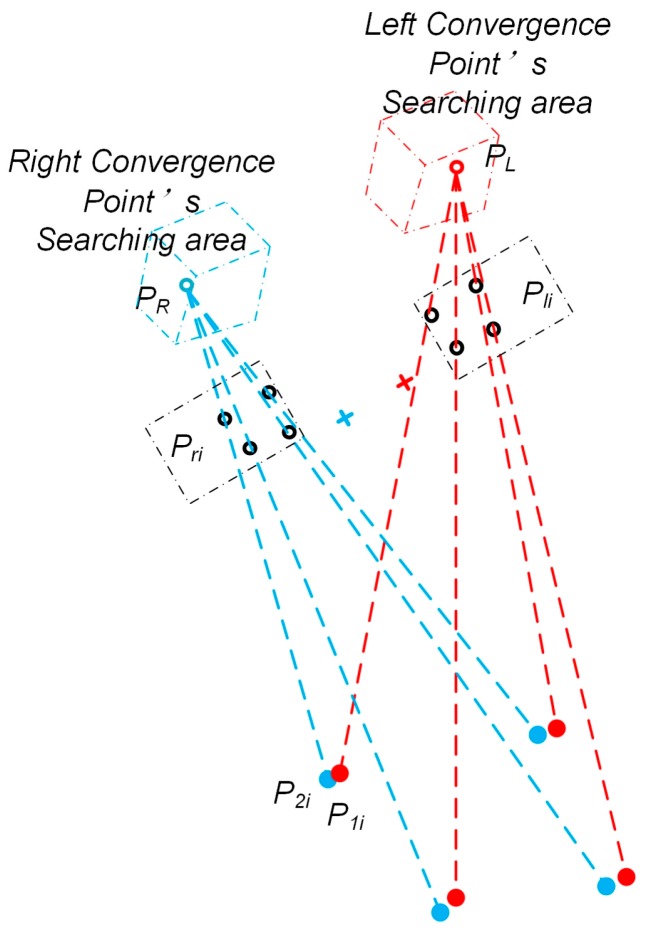
The illustration of the line-of-sight convergence point method and the searching area. *P_li_* and *P_ri_* are the centers of the left and right eyes’ pupils, *P_L_* and *P_R_* are the line-of-sight convergence points, and *P*_1*i*_ and *P*_2*i*_ are the nearest points of two lines which cover *P_L_* and *P_li_*, as well as *P_R_* and *P_ri_*. In our method, *P_L_* and *P_R_* would be searched in restricted areas, while *P*_1*i*_ and *P*_2*i*_ are known.

The studies proposed by [36,37] showed that the comfortable horizontal view range is about 35 degrees, while that of the vertical view range is about 20 degrees. Thus, the work demonstrated in this article limits the view range to the comfortable observation of the eyes. Due to the particularity of the human visual attention mechanism and the error of the line of sight measurement technique, the spatial PoR of the two eyes cannot be obtained directly. In this paper, we suppose that the nearest point of the two lines-of-sight can be used to approximate the spatial PoR. Therefore, this method uses the midpoint of the line which connects the pair of nearest points of the spatial binocular line-of-sight. The spatial contribution of each point is shown in Figure 10.

The central points of the pupils are known as *P_li_* and *P_ri_*, *i* = 1, 2, 3, 4. Assume that there is a pair of line-of-sight convergence points *P_L_* and *P_R_* in the eyeball, in order to calculate the nearest points *P*_1*i*_ and *P*_2*i*_ of the line through *P_li_* and *P_L_* and the line through *P_ri_* and *P_R_*. Let *P_L_* = (*X_L_*, *Y_L_*, *Z_L_*)*^T^*, *P_R_* = (*X_R_*, *YF_R_*, *Z_R_*)*^T^*, *P_li_* = (*X_li_*, *Y_li_*, *Z_li_*)*^T^*, *P_ri_* = (*X_ri_*, *Y_ri_*, *Z_ri_*)*^T^*, *P*_1*i*_ = (*X*_1*i*_, *Y*_1*i*_, *Z*_1*i*_)*^T^*, *P*_2*i*_ = (*X*_2*i*_,*Y*_2*i*_,*Z*_2*i*_)*^T^*. The vertical line between the two lines is subject to:

(3) $$\vec{n}=\vec{P_{1}P_{2}}=\vec{P_{li}P_{L}}\times \vec{P_{ri}P_{R}}$$

The vectors of the two lines are:

(4) $$\begin{array}{l} \vec{P_{li}P_{L}}=\left(X_{L}-X_{li},Y_{L}-Y_{li},Z_{L}-Z_{li}\right)={\left(a_{1i},a_{2i},a_{3i}\right)}^{T}\\ \vec{P_{ri}P_{R}}=\left(X_{R}-X_{ri},Y_{R}-Y_{ri},Z_{R}-Z_{ri}\right)={\left(b_{1i},b_{2i},b_{3i}\right)}^{T} \end{array}$$

so that:

(5) $${\vec{n}}_{i}=\vec{P_{li}P_{L}}\times \vec{P_{ri}P_{R}}={\left(a_{2i}b_{3i}-a_{3i}b_{2i},a_{3i}b_{1i}-a_{1i}b_{3i},a_{1i}b_{2i}-a_{2i}b_{1i}\right)}^{T}$$

According to the property of the nearest point of two spatial lines, two planes which are constituted by the normal vector and these two lines’ vectors are constructed, and the normal vectors of these two planes are:

(6) $$\begin{array}{ll} \vec{n_{i}}\times \vec{P_{li}P_{L}} & =\left(\begin{array}{l} a_{3i}^{2}b_{1i}-a_{1i}a_{3i}b_{3i}-a_{1i}a_{2i}b_{2i}+a_{2i}^{2}b_{1i}\\ a_{1i}^{2}b_{2i}-a_{1i}a_{2i}b_{1i}-a_{2i}a_{3i}b_{3i}+a_{3i}^{2}b_{2i}\\ a_{2i}^{2}b_{3i}-a_{2i}a_{3i}b_{2i}-a_{1i}a_{3i}b_{1i}+a_{1i}^{2}b_{3i} \end{array}\right)\\ & ={\left(k_{1i},k_{2i},k_{3i}\right)}^{T} \end{array}$$

(7) $$\begin{array}{ll} \vec{n_{i}}\times \vec{P_{ri}P_{R}} & =\left(\begin{array}{l} a_{3i}b_{1i}b_{3i}-a_{1i}b_{3i}^{2}-a_{1i}b_{2i}^{2}+a_{2i}b_{1i}b_{2i}\\ a_{1i}b_{1i}b_{2i}-a_{2i}b_{1i}^{2}-a_{2i}b_{3i}^{2}+a_{3i}b_{2i}b_{3i}\\ a_{2i}b_{2i}b_{3i}-a_{3i}b_{2i}^{2}-a_{3i}b_{1i}^{2}+a_{1i}b_{1i}b_{3i} \end{array}\right)\\ & ={\left(k_{4i},k_{5i},k_{6i}\right)}^{T} \end{array}$$

According to the property of two feet on both the normal vector and two lines, we can describe the vectors from the original point to the nearest point as follows:

(8) $$\begin{array}{l} \vec{OP_{1}}=\vec{OP_{L}}+t_{i}\vec{P_{L}P_{li}}\\ \vec{OP_{2}}=\vec{OP_{R}}+{t_{i}}^{\prime }\vec{P_{R}P_{ri}} \end{array}$$

where $\left\{ \begin{array}{l} t_{i}=-\frac{k_{4i}a_{1i}+k_{5i}a_{2i}+k_{6i}a_{3i}}{k_{4i}(X_{L}-X_{ri})+k_{5i}(Y_{L}-Y_{ri})+k_{6i}(Z_{L}-Z_{ri})}\\ {t_{i}}^{\prime }=-\frac{k_{1i}b_{1i}+k_{2i}b_{2i}+k_{3i}b_{3i}}{k_{1i}(X_{R}-X_{li})+k_{2i}(Y_{R}-Y_{li})+k_{3i}(Z_{R}-Z_{li})} \end{array}\right.$.

Thus, the nearest points can be described as:

(9) $$\begin{array}{l} P_{1i}=\left(\begin{array}{l} X_{L}+\frac{k_{4i}a_{1i}^{2}+k_{5i}a_{2i}+k_{6i}a_{3i}}{k_{4i}(X_{L}-X_{ri})+k_{5i}(Y_{L}-Y_{ri})+k_{6i}(Z_{L}-Z_{ri})}\\ Y_{L}+\frac{k_{4i}a_{1i}+k_{5i}a_{2i}^{2}+k_{6i}a_{3i}}{k_{4i}(X_{L}-X_{ri})+k_{5i}(Y_{L}-Y_{ri})+k_{6i}(Z_{L}-Z_{ri})}\\ Z_{L}+\frac{k_{4i}a_{1i}+k_{5i}a_{2i}+k_{6i}a_{3i}^{2}}{k_{4i}(X_{L}-X_{ri})+k_{5i}(Y_{L}-Y_{ri})+k_{6i}(Z_{L}-Z_{ri})} \end{array}\right)\\ P_{2i}=\left(\begin{array}{l} X_{R}+\frac{k_{1i}b_{1i}^{2}+k_{2i}b_{2i}+k_{3i}b_{3i}}{k_{1i}(X_{R}-X_{li})+k_{2i}(Y_{R}-Y_{li})+k_{3i}(Z_{R}-Z_{li})}\\ Y_{R}+\frac{k_{1i}b_{1i}+k_{2i}b_{2i}^{2}+k_{3i}b_{3i}}{k_{1i}(X_{R}-X_{li})+k_{2i}(Y_{R}-Y_{li})+k_{3i}(Z_{R}-Z_{li})}\\ Z_{R}+\frac{k_{1i}b_{1i}+k_{2i}b_{2i}+k_{3i}b_{3i}^{2}}{k_{1i}(X_{R}-X_{li})+k_{2i}(Y_{R}-Y_{li})+k_{3i}(Z_{R}-Z_{li})} \end{array}\right) \end{array}$$

The estimation of the PoR is described as:

(10) $$P_{ei}=(P_{1i}+P_{2i})/2,i=1,2,3,4$$

Before entering the calibration step, we set up a set of conditions to judge whether the values of *P_L_* and *P_R_* are the ideal calibration results. The shape of the calibration target is known, and the best values of *P_L_* and *P_R_* can be found according to whether the distance between the calculated PoR meets the distance between the calibration crosses. After finding suitable values of *P_L_* and *P_R_*, the distance between *P*_1*i*_ and *P*_2*i*_ is minimized to obtain the best *P_L_* and *P_R_* values. This can be described by:

(11) $$({\hat{P}}_{L},{\hat{P}}_{R})=\underset{P_{L}\in A_{init1},P_{R}\in A_{init2}}{\arg\min}\left\{\sum_{i=1}^{3}\left|\left\|P_{e_{i}}-P_{e_{i+1}}\right\|-L\right|+\left|\left\|P_{e_{1}}-P_{e_{4}}\right\|-L\right|\right\}$$

where *L* = 150 mm. The estimation of initial values of *P_L_* and *P_R_* and the set of the initial search area are basic problems. According to the research of the structure and imaging mechanism of the eyeball in [32], we set up the initial values of *P_L_* and *P_R_* at a position of 10 mm away from the center of the four pupil center points. The initial search area is a 4 mm × 4 mm × 4 mm cube centered on the initial coordinates of *P_L_* and *P_R_*. Namely:

(12) $$\begin{array}{l} A_{init1}=\left\{(x,y,z)|x_{cl}-2\leq x\leq x_{cl}+2,y_{cl}-2\leq y\leq y_{cl}+2,z_{cl}-2\leq z\leq z_{cl}+2\right\}\\ A_{init2}=\left\{(x,y,z)|x_{cr}-2\leq x\leq x_{cr}+2,y_{cr}-2\leq y\leq y_{cr}+2,z_{cr}-2\leq z\leq z_{cr}+2\right\} \end{array}$$

Among them, $(x_{cl},y_{cl},z_{cl})=\left(\frac{1}{4}\sum_{i=1}^{4}X_{li},\frac{1}{4}\sum_{i=1}^{4}Y_{li},\frac{1}{4}\sum_{i=1}^{4}Z_{li}+10\right)$, and $(x_{cr},y_{cr},z_{cr})=\left(\frac{1}{4}\sum_{i=1}^{4}X_{ri},\frac{1}{4}\sum_{i=1}^{4}Y_{ri},\frac{1}{4}\sum_{i=1}^{4}Z_{ri}+10\right)$.

According to the calculation Equation of *P_ei_*, it can be found that the function is not a convex function and the global optimal solution cannot be found by convex optimization. Therefore, this paper sets up several step lengths to search for the position of possible line-of-sight convergence points several times. A large step length is used first to search for the best *P_L_* and *P_R_* values at the current step. A small step length and a small search area is then used to find the new best *P_L_* and *P_R_* values. Cycle this process several times, a pair of more accurate line-of-sight convergence points can be determined. The step length is equivalent to the subdivision of the search area. For example, when the search range is a 4 mm × 4 mm × 4 mm cube and the search step length is 1 mm, the number of all search points is (4 + 1)^3^ = 125. The initial step length is set to 0.2 mm. In this method, each search step length is half of the previous search step length, and the search area range is also half of the previous search range. When the search step length is less than 0.0005 mm, the search process ends.

In addition to calculating whether the distance between the PoR meets the positional relationship between the calibration crosses, a more accurate line-of-sight convergence point can be obtained by calculating the nearest distance between the lines of sight. Namely:

(13) $$({\hat{P}}_{L},{\hat{P}}_{R})=\underset{P_{L}\in A_{cur1},P_{R}\in A_{cur2}}{\arg\min}\left\{\sum_{i=1}^{4}\left\|P_{1i}-P_{2i}\right\|\right\}$$

**Figure 11:**
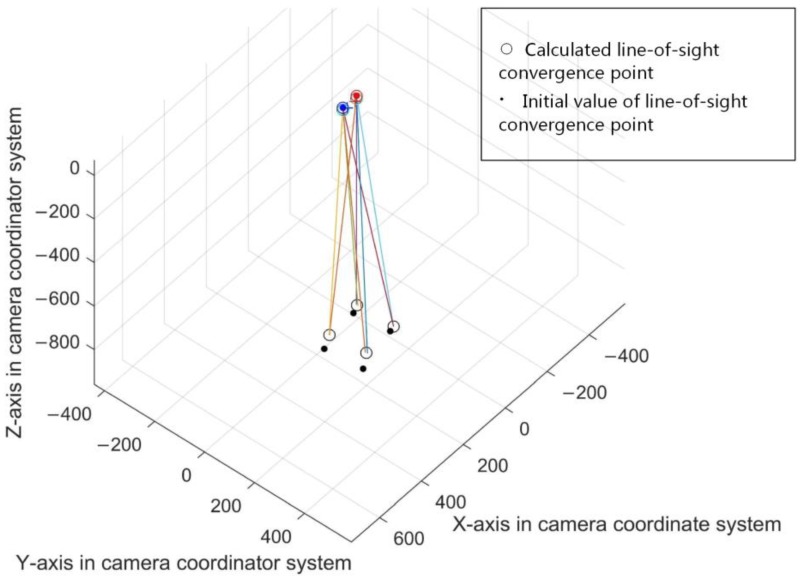
The calibration result in one step.

However, we need to unify the two judgment bases, so this paper proposes a function to merge these two judgment bases:

(14) $$({\hat{P}}_{L},{\hat{P}}_{R})=\underset{P_{L}\in A_{cur1},P_{R}\in A_{cur2}}{\arg\max}\left(\exp\left(\begin{array}{l} -\frac{\sum_{i=1}^{4}\left\|P_{1i}-P_{2i}\right\|}{\sqrt{\frac{1}{n-1}\sum_{j=1}^{n}{\left(\sum_{i=1}^{3}\left|\left\|P_{e_{j,i}}-P_{e_{j,i+1}}\right\|-L\right|+\left|\left\|P_{e_{j,1}}-P_{e_{j,4}}\right\|-L\right|\right)}^{2}}}\\ -\frac{1}{\varepsilon }\sum_{i=1}^{3}\left|\left\|P_{e_{i}}-P_{e_{i+1}}\right\|-L\right|+\left|\left\|P_{e_{1}}-P_{e_{4}}\right\|-L\right| \end{array}\right)\right)$$

where *n* is the number of search points in the current search area and *ε* is the sensitivity coefficient, in this article ε=10. The calibration result in one step is shown in Figure 11.

To summarize the algorithm:

1. First set an initial value of *P_L_* and *P_R_*, the initial search areas *A_init1_* and *A_init2_*, and the search step length *st*.
2. Calculate the judgment value of each search point according to Equation (14) and take the best *P_L_* and *P_R_* values in the current search area as the center point of the next search.
3. The current search area *A_cur_*_1_, *A_cur_*_2_ and the search step length *st* are redefined, wherein the boundary width of *A_cur_*_1_ and *A_cur_*_2_ is half of that in the previous search, and the value of *st* is also half of that in the previous search.
4. Repeat step (2) to obtain the best *P_L_* and *P_R_* values in the current search area and use them as the center of next search area for the next search.
5. Repeat step (3). If *st* < 0.0005 mm, the calculation is finished, and current *P_L_* and *P_R_* values are the final calculation results. If not, repeat step (2).

#### 3.3.2. Coordinate Alignment and Line-of-Sight Convergence Point Fitting Method

**Figure 12:**
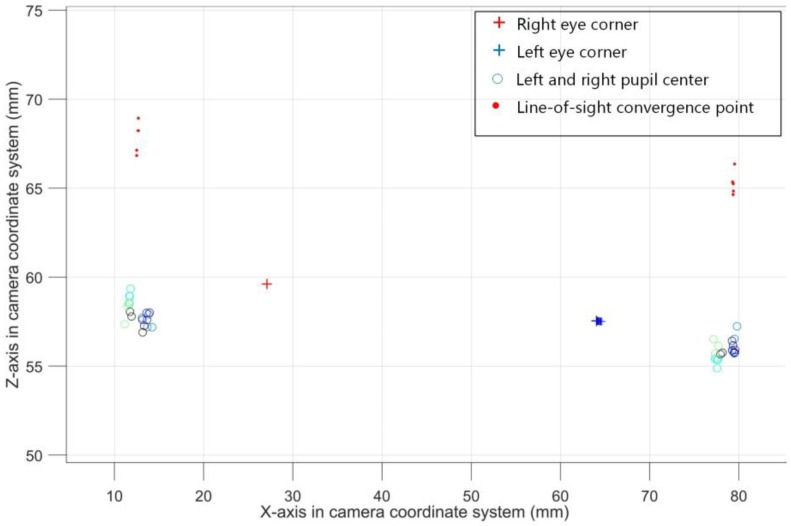
The distribution of the line-of-sight convergence points which are solved in the calibration process, and these points are aligned by the inner eye corner vector.

After obtaining several pairs of line-of-sight convergence points, comparing with the pupil size, we can find this rule: when the pupil is larger, the line-of-sight convergence point is closer to the pupil; when the pupil is smaller, the line-of-sight convergence points is far away from the pupil. In addition, after obtaining multiple pairs of line-of-sight convergence points and corresponding pupil sizes, there is the possibility of correlating the spatial coordinates of the line-of-sight convergence points with the inner eye corners. However, before that, it is necessary to establish an eye coordinate system based on the spatial coordinates of the inner eye corners, so that the relationship between each feature point can be described at any time. In Figure 12, the calibrated line-of-sight convergence points which have been aligned in eye coordinate system are shown. We can see the distribution of these points.

Before analyzing the relationship between the spatial coordinates of the line-of-sight convergence point and the pupil size, we first determine the relationship between the line-of-sight convergence point and the inner eye corner. As a stable facial feature, the inner eye corner can be used as a reference for a facial pose. However, only one pair of points cannot restrict all degrees of freedom of the facial pose. Further, there is no restriction on head pitch. The device described in this paper restricts the pitch motion of the head when in use, greatly inhibiting the influence of the change of the head pitching attitude. Thus, the line-of-sight convergence point can be used as a reference for the estimation of the PoR.

**Figure 13:**
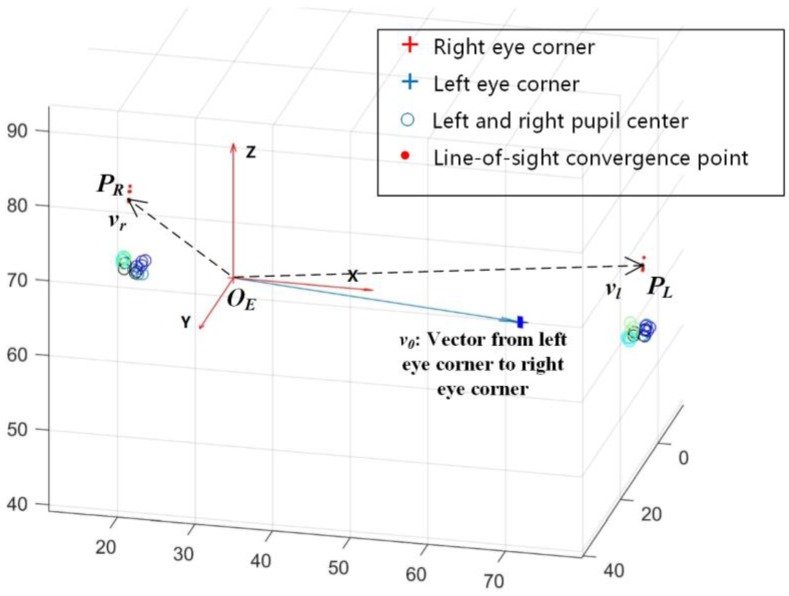
The origin of the eye coordinate system is built on the right eye corner point; the axis direction and the scale factor are consistent with the camera coordinate system. The blue arrow shows the inner eye corner vector from the left eye corner to the right eye corner. When the position and posture of the face changes, the position of the line-of-sight convergence point can be obtained by calculating the rotation and translation matrix between the new inner eye corner vector and the initial reference in the calibration result.

In this paper, the eye coordinate system is established with the right inner eye corner as the origin, as shown in Figure 13. And its coordinate axis direction and scale factor are consistent with the camera system coordinate system. At the same time, the left and right eye corners are connected to form the vector of the inner corner of the eye. This vector can be used as a reference to describe the coordinates of the line-of-sight convergence points so as to make use of the calibration results. In this paper, the direction and length of the vectors from the origin of eye coordinate system to *P_L_* and *P_R_* are used to describe the spatial coordinate of the line-of-sight convergence point.

In the eye coordinate system, *v*_0_ is the inner eye corner vector as the initial reference for aligning the other inner eye corner vectors, and the right corner coordinate is the initial right corner. *v_r_* is the vector from the origin of the eye coordinate system to the right line-of-sight convergence point. *v_l_* is the vector from the origin of the eye coordinate system to the left eye line-of-sight convergence point. Thus, we can obtain:

(15) $$\begin{array}{l} \vec{v_{l}}=R_{l}\vec{v_{0}}=\vec{P_{L}O_{E}}\\ \vec{v_{r}}=R_{r}\vec{v_{0}}=\vec{P_{R}O_{E}} \end{array}$$

*R_l_* and *R_r_* are the rotation matrixes from *v*_0_ to *v_l_* and *v*_0_ to *v_r_*. Meanwhile, the mode lengths of *v_r_* and *v_l_*, $\left\|\vec{v_{r}}\right\|,\left\|\vec{v_{l}}\right\|$, are also calculated.

When the inner eye corner vector *v_i_* is in a different position and direction compared to *v*_0_, we first translate *v_i_* from the camera coordinate system to the eye coordinate system defined by *v*_0_. Then, the right corner coordinate is aligned with the initial right corner coordinate by a translation vector *T’*. Then the rotation matrix *R’* between *v*_0_ and *v_i_* can be calculated: $\vec{v_{i}}=R^{\prime }\vec{v_{0}}$.

The line-of-sight convergence point vectors in the eye coordinate system in which *v_i_* is located is:

(16) $$\vec{v_{r}^{\prime }}=R_{r}\vec{v_{i}}=R_{r}R^{\prime }\vec{v_{0}},\vec{v_{l}^{\prime }}=R_{l}\vec{v_{i}}=R_{l}R^{\prime }\vec{v_{0}}$$

Therefore, *P_L_* and *P_R_* can be calculated:

(17) $$\begin{array}{l} P_{R}=R_{r}R^{\prime }\vec{v_{0}}\frac{\left\|\vec{v_{r}}\right\|}{\left\|\vec{v_{r}^{\prime }}\right\|}+T^{\prime }\\ P_{L}=R_{l}R^{\prime }\vec{v_{0}}\frac{\left\|\vec{v_{l}}\right\|}{\left\|\vec{v_{l}^{\prime }}\right\|}+T^{\prime } \end{array}$$

After determining the spatial position relationship of the line-of-sight convergence point with respect to the inner eye corners, we use polynomial fitting based on the least squares method to determine the relationship between the line-of-sight convergence point and the pupil size. Through experiments, it is found that the polynomial fitting with the highest term being quadratic term can be effective. The obtained line-of-sight convergence point and inner eye corner are converted to the initial inner eye corner vector reference, and the distribution of the line-of-sight convergence points is observed and found to be basically along a straight line perpendicular to the inner eye corner vector. Therefore, we separate the *X*, *Y*, and *Z* coordinates of the line-of-sight convergence point, and firstly determine the fitting equation of *Z* and the pupil size *S*. Then the spatial line equation is used to fit the relationship between *X* and *Z*, *Y* and *Z* respectively. Because *Z* coordinate is sensitive to the pupil size *S*, we first establish a fitting equation between *Z* and *S*:

(18) $$Z=\gamma_{0}+\gamma_{1}S+\gamma_{2}S^{2}$$

While the Equation of the spatial straight line can be written as follows:

(19) $$\frac{X-X_{0}}{m}=\frac{Y-Y_{0}}{n}=\frac{Z-Z_{0}}{p}$$

Therefore, Equations of *X* and *Y* can be obtained:

(20) $$\left\{ \begin{array}{l} X=\frac{m}{p}Z+\left(X_{0}-\frac{m}{p}Z_{0}\right)\\ Y=\frac{n}{p}Z+\left(Y_{0}-\frac{n}{p}Z_{0}\right) \end{array}\right.$$

Finally, the fitting parameter Equation is:

(21) $$\left\{ \begin{array}{l} Z=\gamma_{0}+\gamma_{1}S+\gamma_{2}S^{2}\\ X=\alpha_{0}+\alpha_{1}Z\\ Y=\beta_{0}+\beta_{1}Z \end{array}\right.$$

The calibration process finally obtains the relationship between the pupil size and the spatial coordinates of the line-of-sight convergence point within the initial inner eye corner vector reference. When in use, the spatial coordinates of the line-of-sight convergence point within the initial inner eye corner vector reference can be obtained by inputting the pupil size into the parametric equation, and the output spatial coordinates can be further converted to the new spatial coordinates within the actual inner corner vector reference.

## 4. Experiments

The pupil size changes with the change of human consciousness, so the coordinates of the calculated Point-of-Regard (PoR) have a large distribution. Therefore, the experiment in this paper uses the average of the coordinates of the PoR within a period of time to represent the measured PoR. In addition to counting the average error of the measurement results, this paper also compares the shape of the object being watched with the position of the PoR in the test. The difference between the estimated 3D PoR and the spatial coordinates of the gaze point in the real is used to evaluate the effectiveness of the proposed method.

The experiment was conducted in a fixed indoor illumination condition. Images of about 0.5 s were captured at each fixation. The experimental results are all in the system camera coordinate system.

### 4.1. Intuitive 3D Point-of-Regard Estimating Experiments

**Figure 14:**
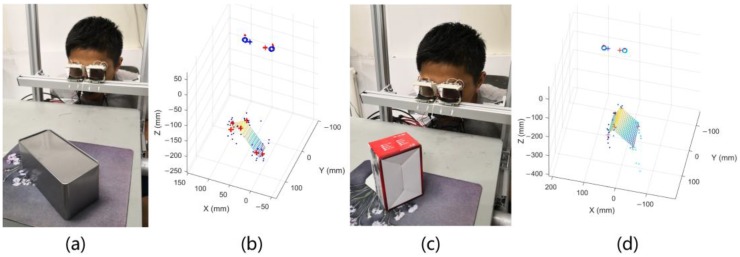
Placing two different boxes in front of the gaze tracking system and allowing the person to look at the six visible corners on the box. (**a**) and (**c**) show the real pictures when a person was looking at the visible corners on the box through the system. (**b**) and (**d**) show the solving result of the corners on the box. The figure shows that this method can output a more accurate response to the stereoscopic perception of human vision.

In this part, a person completes a calibration process in the indoor environment and then involves in two groups of experiments of watching different objects. Firstly, an actual object was used to test the result: square boxes with different shapes were placed at different positions so that the person could look at the six visible corners in the field of view to take the measurement results. The results are shown in Figure 14.

**Figure 15:**
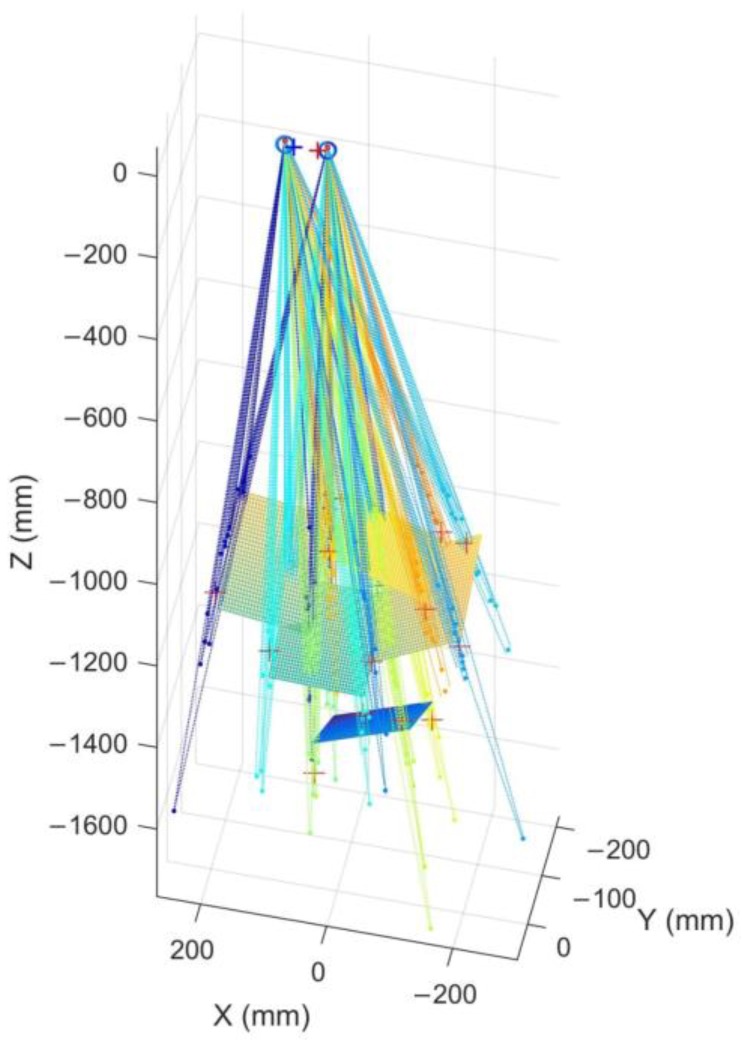
The graph shows the experimental result when the tester is watching the calibration board at five different distances.

Secondly, the calibration board was then used to test the extraction of depth perception: the calibration board is placed at different positions 1 m away from the system, the test point was selected for a person to watch, and the obtained PoR with the position on the test point was compared. This experiment can further prove the relationship between the depth perception of the human vision system and the pupil size under fixed illumination conditions. The results are shown in Figure 15.

### 4.2. Influences of Different People or in Different Illumination Condition

As a general method, the 3D PoR estimation model needs to meet the needs of measuring PoR of different people. Another item that needs to be improved in this method is the influence of pupillary light reflection on the calculated convergence point due to changes in environmental illumination. This Section describes these two verification experiments together. We selected the testing time at night, the indoor lighting includes several sets of daylight lamps and all of them could be controlled by switches. We invited nine testers, and each of them was allowed to complete the calibration process once under a fixed illumination condition, with a fixed calibration board was placed at a position 1 m away. Let the testers look at the four test points on the calibration board, then adjusted the condition of indoor illumination several times and compared the estimating 3D PoR.

**Figure 16:**
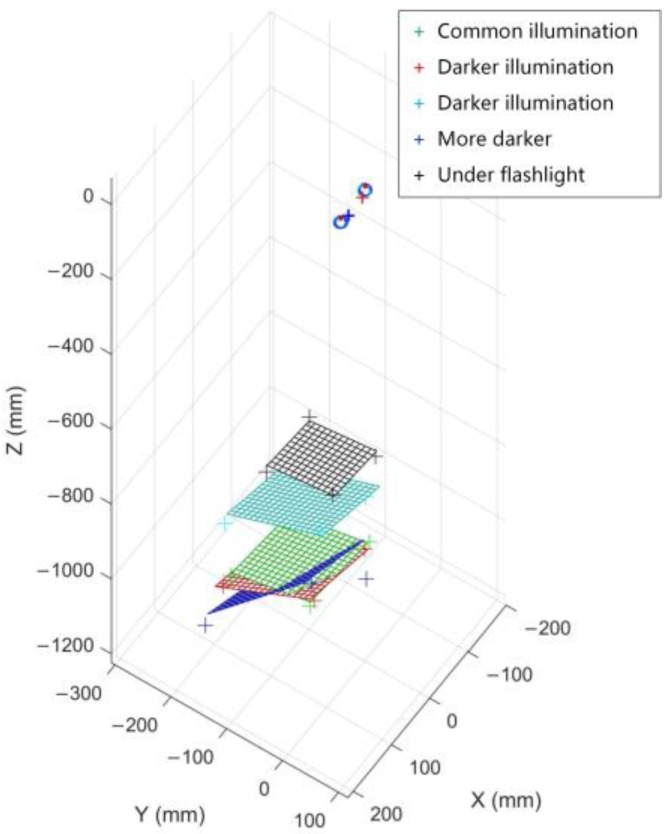
The graph shows the experimental result when the tester was watching the calibration board in five different illumination conditions.

The experiment can be divided into two parts:

1. The illumination condition of this experiment was the same as that of the calibration process. We invited nine testers to look at the calibration board respectively. Each one was allowed to participate in one group of experiments, and there were eight repeated experiments in each group. The interval between each experiment were 30 s. According to the distribution of the solved 3D PoR in each group, the average Cartesian error of every tester’s 3D PoR was listed in Table 1.
2. We let a tester look at the calibration board under different lighting conditions and observe the distribution of 3D PoR. During the experiment, the indoor illumination changed from common to dark for four times. After adjusting to the dark, we used a flashlight to illuminate the calibration board, and then took one more experiment to observe the experimental results. The intuitive experimental result is shown in Figure 16.

These two experiments show that the 3D PoR distribution errors of different testers are approximate and concentrated in the space near the actual observed points. In experiments under different lighting conditions, we can find that the 3D PoR test results are adjacent when the lighting conditions are close to the condition during the calibration. However, when the intensity of light decreases, the test results become unpredictable. This instability can reflect that the depth perception of human vision is inaccurate in dark places. When the daylight lamps are all turned off and within a flashlight illuminating, the result is the same as the shape of the calibration board, but the solved spatial distribution of 3D PoR is far away from the actual position of the calibration board. Similarly, it can be considered that the method in this paper is not applicable when looking at luminous objects such as monitors.

### 4.3. Error of the Method in Different Distances

Finally, we test the accuracy of the spatial gaze point coordinates under different distances. A necessary experimental environment is constructed. The test object is the calibration board used in the calibration process. We fix a calibrated camera as a test camera under the gaze tracking system so that the tester can first look at a closer calibration board. At the same time, the test camera is used to solve the spatial coordinates of the calibration board, and then the gaze tracking system and the test camera are associated. In this way, we can obtain the space coordinates of the calibration board under the camera coordinate system of the gaze tracking system.

In the experiment, a target was fixed to seven different distances. At each distance, the tester carried out 15 repeated experiments, and then we counted the average errors of the coordinates of the PoR. We also compared the 3D PoR estimation result in [30], and this experimental result is obtained with indoor lighting condition. The experimental results are shown in Table 2.

According to the experimental result shown in Table 2, the 3D PoR estimation of our method is not accurate as the compared method. The errors on the *X* and *Y* can be controlled to a low level, while the errors on the *Z* is much bigger. However, within a relatively simplified gaze estimation model and a fast calibration process, the actual performance of the proposed method is still in line with the expectation.

### 4.4. Discussion

We employed the test camera for capturing scene images to check the difference between the estimated 3D PoR and the ground truth. According to the experimental result, our method can accurately estimate the spatial coordinates of the object of interest in indoor environment. As we predicted, lighting condition changes influence the 3D PoR estimation. This is because the lighting condition during calibration process is different from that when applying, which is a defect of this method. However, this method can also show strong adaptability when used indoors. In addition, the accuracy of this method should be improved, but using a simplified calibration process may be difficult to further improve the accuracy. This is also the focus of future research.

## 5. Conclusions

We propose a novel approach to estimate 3D Point-of-Regard in free space, which can be used as a specific head-mounted multi-camera gaze tracking device. Based on the pupillary accommodation reflection property in the human visual system, this method can perceive the depth perception of human vision, and takes the fixation point at the center of the view range of both eyes as the auxiliary assumption in fixation duration, and further associates the line-of-sight convergence point with the pupil size to form a model for measuring the 3D PoR. In addition, this paper proposes a gaze-tracking device which combines four cameras and semi-transparent mirrors, and also uses a novel near-infrared lighting source to enhance the contrast of pupil images. The pupil center and the inner eye corner are the basic features of the line-of-sight measurement. Through a simple calibration process, the line-of-sight convergence point is searched and the corresponding pupil size is correlated. The parameters of the whole line-of-sight measurement model are very simple, and the center point of the nearest points of the spatial line-of-sight of the left and right eyes is represented as the measured PoR. The method proposed in this paper is examined through several experiments: it can be proved that the method can be used to measure the spatial coordinates of PoR, and that the gaze-tracking device proposed in this paper is a novel method for wearable devices. Experimental results show that the gaze point measurement model proposed in this paper has sufficient measurement accuracy, while the model and calibration process are relatively simple, and they can reflect the depth perception information of human vision in fixed lighting conditions.

## Figures and Tables

**Table 1:** Average errors of 3D PoR estimation in centimeters of every tester.

Unit: cm	1	2	3	4	5	6	7	8	9
**Error on *X***	0.2 ± 0.2	0.4 ± 0.2	0.4 ± 0.4	0.4 ± 0.3	0.4 ± 0.3	0.3 ± 0.2	0.4 ± 0.3	0.4 ± 0.2	0.5 ± 0.4
**Error on *Y***	0.4 ± 0.3	0.6 ± 0.3	0.6 ± 0.5	0.5 ± 0.4	0.6 ± 0.5	0.5 ± 0.4	0.6 ± 0.5	0.5 ± 0.5	0.5 ± 0.5
**Error on *Z***	3.8 ± 2.6	4.8 ± 2.9	5.1 ± 3.1	4.3 ± 2.8	5.1 ± 3.4	4.1 ± 3.0	4.8 ± 3.1	4.6 ± 3.1	5.3 ± 4.0
**Overall**	3.8 ± 2.6	4.9 ± 2.9	5.2 ± 3.1	4.3 ± 2.8	5.1 ± 3.4	4.2 ± 2.9	4.9 ± 3.1	4.7 ± 3.1	5.3 ± 3.9

**Table 2:** Average errors in centimeters measured as the difference between the estimated PoR by the proposed method and the 3D coordinates of the calibration target solved by the test camera.

Unit: cm	0.8 m	1.5 m	2 m	2.5 m	3 m	4 m	6 m
**Error on *X***	0.4 ± 0.5	0.6 ± 0.5	0.7 ± 0.6	1.1 ± 0.6	0.9 ± 0.5	1.3 ± 0.6	2.3 ± 0.8
**Error on *Y***	0.5 ± 0.5	0.8 ± 0.7	0.6 ± 0.4	0.8 ± 0.6	0.8 ± 0.5	1.3 ± 1.1	3.0 ± 0.4
**Error on *Z***	5.2 ± 3.1	6.1 ± 3.8	8.3 ± 6.0	9.6 ± 7.1	13.8 ± 7.8	13.3 ± 10.5	23.7 ± 12.5
**Overall**	5.3 ± 3.3	6.3 ± 4.3	8.4 ± 5.9	9.9 ± 7.0	13.9 ± 7.7	14.5 ± 10.9	24.3 ± 12.5
**Overall in** [30]	2.1 ± 0.3	/	3.6 ± 0.3	/	5.7 ± 0.5	/	16.9 ± 1.2
